# The challenge of exploring neuropsychiatric and cognitive symptoms after spontaneous intracerebral hemorrhage

**DOI:** 10.1111/ene.16585

**Published:** 2024-12-18

**Authors:** Giuseppe Scopelliti, Charlotte Cordonnier, Leonardo Pantoni

**Affiliations:** ^1^ Neurology Unit Luigi Sacco University Hospital Milan Italy; ^2^ Univ. Lille, Inserm U1172 – Lille Neuroscience & Cognition CHU‐Lille Lille France; ^3^ Department of Biomedical and Clinical Sciences, Neuroscience Research Center University of Milan Milan Italy; ^4^ Department of Neurorehabilitation Sciences Casa di Cura Igea Milan Italy

Spontaneous intracerebral hemorrhage (ICH) is an extremely dangerous condition, with overall greater social‐economic burden than ischemic stroke despite lower incidence. Approximately half of patients with acute ICH do not survive beyond the first year, and those who do often face significant, lasting disability [1]. Qiuyi Jiang et al. investigated one of the more subtle long‐term effects of ICH, the affective and cognitive symptoms that have been often overlooked by stroke neurologists, but are an especially pressing burden for patients and their caregivers [2, 3]. This study, conducted in China, included nearly 1600 patients, marking the largest cohort on this topic to date. However, the majority of participants had deep ICH (85%) and were relatively young (median age 57 years), limiting the generalizability of the findings to older populations with lobar ICH.

Although the study explicitly focused on “small” ICHs (median volume of 10 mL), it should be noted that the majority of previous studies on long‐term cognitive and neuropsychiatric outcomes of ICH have also predominantly included patients with smaller hematoma volumes [4, 5]. This is mainly because larger hemorrhages are associated with poor short‐term survival, or patients are less likely to be able to attend outpatient clinic for long term follow‐up. Similar considerations can be made regarding studies on the long‐term cognitive consequences of ischemic stroke, in which patients with more severe events and language impairment are often excluded, resulting in an unavoidable bias in evaluating long‐term trajectories [6]. Nevertheless, we might hypothesize that those patients suffering from lower levels of disability after the index event are probably the ones who are going to benefit the most from a long‐term stroke neuropsychiatric and cognitive follow‐up.

Given that most studies have predominantly focused on small ICHs, our understanding of the cognitive and neuropsychiatric consequences of ICH survivors so far may reflect more the influence of the underlying cerebral small vessel disease (e.g., arteriolosclerosis and cerebral amyloid angiopathy) rather than the direct impact of the initial ICH event. Although this study did not assess MRI features of cerebral small vessel disease, the authors found that potentially modifiable factors related to ICH at baseline (e.g., blood pressure levels, length of hospitalization, and anteroposterior diameter of the index ICH) could influence long‐term cognitive and affective outcomes. This finding underscores the importance of consistently including cognitive and affective measures as outcome variables in clinical trials of acute phase interventions.

Another important catch on the current topic is the complex presentation of affective symptoms following an ICH, and, more broadly, in patients with cerebrovascular diseases. Qiuyi Jiang and colleagues adopted a pragmatic approach, using both in‐hospital and telephone‐based assessments to evaluate patients' cognitive and affective status. While this method may boost neuropsychiatric screening rates, categorizing a patient as simply having depressive or anxiety symptoms may not capture the full complexity of the neuropsychiatric profile, especially as growing evidence shows that different mood and cognitive manifestations are often interlinked and comorbid. We now recognize that the neuropsychiatric profile may reflect both the structural integrity and age‐related changes of the brain (e.g., neurodegeneration, vascular burden), offering insights into the cognitive status of patients with stroke [5, 7]. This understanding is especially important when evaluating post‐ICH depression, as different neuropsychiatric patterns may be present under the general category of depressive symptoms; for example, identifying apathetic traits alongside mood disturbances could help to pinpoint the patients at greater risk for cognitive impairment [8]. The tools currently used in clinical practice do not adequately capture the nuanced neuropsychiatric status of cerebrovascular patients: developing new, multidimensional screening instruments to better assess this complexity remains a significant challenge for the future.

## AUTHOR CONTRIBUTIONS

**Giuseppe Scopelliti:** Conceptualization; writing – original draft. **Charlotte Cordonnier:** Writing – review and editing; conceptualization. **Leonardo Pantoni:** Writing – review and editing; conceptualization.

## CONFLICT OF INTEREST STATEMENT

None.

## Data Availability

Data sharing not applicable to this article as no datasets were generated or analysed during the current study.
